# Treatment of persistent congenital chylothorax with intrapleural injection of sapylin in infants

**DOI:** 10.12669/pjms.325.10142

**Published:** 2016

**Authors:** Qing-Wang Hua, Zhi-Yong Lin, Xing-Ti Hu, Qi-Feng Zhao

**Affiliations:** 1 Qing-Wang Hua, MD. The Children’s Department of Cardiovascular & Thoracic Surgery, Childern’s Heart Center, The 2nd Affiliated Hospital & Yuying Children’s Hospital of Wenzhou Medical University, Wenzhou, P.R. China, 325000; 2 Zhi-Yong Lin, MD. The Children’s Department of Cardiovascular & Thoracic Surgery, Childern’s Heart Center, The 2nd Affiliated Hospital & Yuying Children’s Hospital of Wenzhou Medical University, Wenzhou, P.R. China, 325000; 3 Xing-Ti Hu, PhD. The Children’s Department of Cardiovascular & Thoracic Surgery, Childern’s Heart Center, The 2nd Affiliated Hospital & Yuying Children’s Hospital of Wenzhou Medical University, Wenzhou, P.R. China, 325000; 4 Qi-Feng Zhao, MD. The Children’s Department of Cardiovascular & Thoracic Surgery, Childern’s Heart Center, The 2nd Affiliated Hospital & Yuying Children’s Hospital of Wenzhou Medical University, Wenzhou, P.R. China, 325000

**Keywords:** Chylothorax, Chemical pleurodes, Infant, Sapylin

## Abstract

Test the therapeutic efficacy of Sapylin in resolving persistent Congenital Chylothorax (CC) in four infants who failed to respond to conservative medical therapy including Erythromycin and/or Octreotide management. All cases were cured and have no adverse reactions during follow-up. The result shows Sapylin is effective in reducing chylous production.

## INTRODUCTION

Congenital chylothorax (CC) is a rare disease and a cause of respiratory distress with major nutritional and immunological consequences. Therefore, it is critical to decrease pleural effusion promptly. Management of CC necessitates a multidisciplinary approach. Conservative management prior to surgical approach in the treatment of chylothorax cases.

The common conservative treatment for persistent CC is often futile. Recently, octreotide and chemical pleurodesis were used to treat the persistent CC. However, the systematic study in literature for octreotide therapy is limited and the efficacy of octreotide treatment is vary. In addition, octreotide has many side effects. Chemical pleurodesis is a method to treat CC through injecting the chemical agent into chest, which could induce the chemical pleural inflammation and adhesions, prompt the pleural cavity closure and prevent the chyle leakage. The analogs of Sapylin, OK-432 was used for malignant pleural effusion and lymphatic tumor treatment in the past.^1^,^2^ Here, we report the therapeutic effect of intrapleural instillation of Sapylin on persistent CC in four cases.

## CASE REPORT

The 2nd Affiliated Hospital & Yuying Children’s Hospital of Wenzhou Medical University Institutional Review Board approved this investigation and informed consents were obtained from the parents of all the infants in this study. All procedures performed in the study involving human participants were in accordance with the ethical standards of the institutional and/or national research committee and with the 1964 Helsinki Declaration and its later amendments or comparable ethical standards. A retrospective review was performed, examining the medical records of all kids who had received Sapylin (Group A Streptococcus Preparation, 1KE, Shandong Lukang Pharmaceutical Group Luya Co. Ltd, China) intrapleural injection for the treatment of persistent CC over a 43-month period between 2011 and 2014. Diagnosis of chylous effusion in infants was based on findings of fluid with a milk-like appearance, a concentration of triglycerides in pleural effusion >1.1 mmol/L, and a total cell count > 1000 cells/microL, with a lymphocyte fraction > 80%. The bacterial culture of effusion was sterile.

Baseline patient characteristics and the treatment data in four infants are listed in Table-I. Sapylin treatment was identified in patients who met our inclusion criteria, which was resistant to mainly conservative treatment including Erythromycin and/or Octreotide therapies in three weeks. The drainage tube was closed for 6-8 hour after Sapylin dissolved in 10ml of 10% glucose solution was injected into thoracic cavity. Clinical cure was defined as a decrease in chylothorax drainage < 5mL/kg/day and resolution of the chylothorax. There was no mortality and all patients survived.

**Table-I:** Patient baseline characteristics and treatment data of congenital chylothorax (n=4)

	*Patient No.1*	*Patient No.2*	*Patient No.3*	*Patient No.4*
Sex	M	M	F	M
Age	2mos	1mos	1mos	16dys
Gestation (wks)	38	40	38	39
Birthweight (g)	3500	3450	3050	3260
Hydrops/Location	Yes/Left	Yes/Left	Yes/Right	Yes/Left
Genetic/anomalies	-	-	-	-
Ventilation	+	-	-	-
Hypoalbuminaemia	+	+	+	+
Total duration of chest drains (dys)	53	31	32	35
The maximum daily drainage (mL)	350	210	185	156
Treatment with Octreotide	No	Yes	Yes	Yes
Treatment with Erythromycin	Yes	Yes	Yes	Yes
Operation	Yes	No	No	No
The initial treatment time of Sapylin (after admission)	34th	22th	24th	28th
Frequency of Sapylin	2	2	2	2
The dose of Sapylin (KE)	0.5,0.75	0.5,0.5	0.5,0.5	0.5,0.5
Associated complications	ARDS	-	-	-
Resolutionn with Sapylin	Yes	Yes	Yes	Yes
Duration of hospital stay (dys)	77	42	41	45
Outcome	Discharge	Discharge	Discharge	Discharge

**Fig.1:**
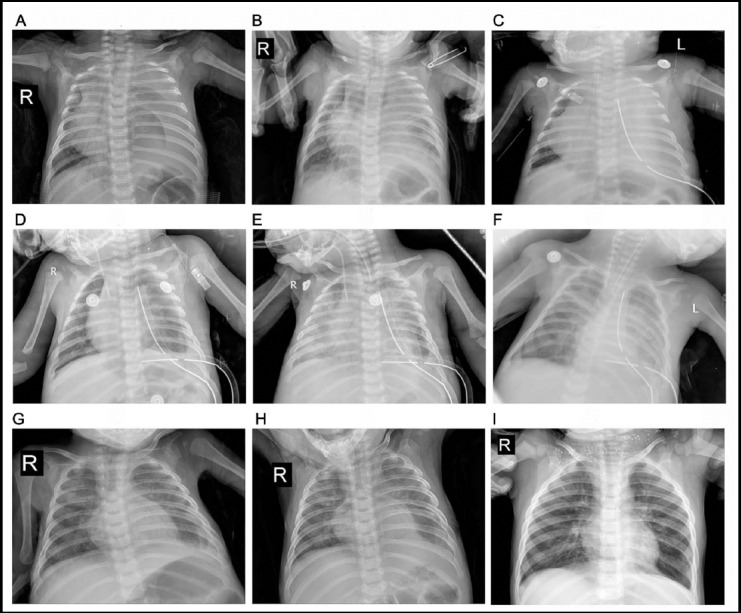
The clinical course of treatment with Sapylin manifestating on CXR (A~I) in a sample responder (patient 4). A. Admission; B. After three weeks of conservative treatment; C. After ligation of thoracic duct; D. Another chest tube was placed, E. ARDS after sapylin was given; F. ARDS was relieved after 1 week; G. After weaning from the ventilator and taking off the chest tube; H. A month after discharge; I. 1 year after discharge.

The clinical course of treatment with Sapylin manifestating on CXR (A~I) (Patient No. 1) is shown in Fig.1. Diagnosis and treatment of this patient was as follows: After the admission diagnosis of CC, we carried out the thoracic drainage and total parenteral nutrition, but the chest drainage was not reduced. Injection of erythromycin into thoracic cavity was still invalid and we performed thoracotomy and ligation of thoracic duct after about 4 weeks of admission. We added a drainage tube after operation because of wrapped effusion, but the amount of fluid did not reduce. We injected Sapylin into pleural cavity in the postoperative day of 7, 8 and the acute respiratory distress syndrome (ARDS) occurred after 3 days. The SPO_2_ maintains through the way of endotracheal intubation and mechanical ventilation, and raise up to 90%(>) after plus PEEP and inhaling nitric oxide. After one week, the ARDS was alleviated and the chest drainage was significantly reduced. The patient finally cured after 1 month.

The emergence of ARDS due to using Sapylin for two consecutive days in patient No 1. Fortunately, the patient was out of danger after treatment. It also provides clinical experience for future treatment. In patient No 2~4, Sapylin was used twice but within intervals of three days. The dosage was always 0.1-0.2KE/kg/day. The result shows no side effect was observed in these cases. After discharge, four patients were traced for follow-up from 6 months to two years without recurrence, and all of them achieved age appropriate developmental milestones.

## DISCUSSION

The treatment of persistent CC is still a challenge in clinic.^3^ There is no standard choice between conservative treatment and surgery. Early surgical interventions or conservative treatment is still worth discussing. Surgery may lead to a high number of thoracotomies and conservative treatment certainly increase hospitalization time.^4^,^5^

Recently, there are few studies about octreotide treatment in CC, but the effects were reported inconsistently. Many case reports have shown octreotide is safe without side effect.^6^ However, Maayan-Metzger et al.^7^ reported that somatostatin treatment of CC may induce transient hypothyroidism in newborns. No practice recommendation could be made based on current limited studies. In our study, three patients treated with octreotide did not achieve the expected effect in 1-2 weeks, although it may be related to the cases we selected. Nevertheless, it indicated the effectiveness of Octreotide is not as high as it has been reported.

Chemical pleurodes is an efficient way to treat CC. Under conditions of the persistent and refractory cases, even post-operation, pleurodesis might be the only method, like Riquet et al.^8^ reported. Deurloo et al.^9^ even evaluated the effect of prenatal therapeutic interventions on perinatal outcome in pregnancies complicated by isolated fetal hydrothorax with hydrops, and found pleurodesis is worth to be recommended.

Sapylin, the new medicine, may facilitate pleurodesis by activation of natural killer cells and T cell-inducing cytokine production, stimulating fibrin adhesion, and producing a strong inflammatory response. Four patients who failed to respond to traditional conservative medical therapy were treated with Sapylin, and all cases were cure. The result shows Sapylin was more effectively than Erythromycin and/or Octreotide. This treatment may reduce the need for surgery significantly, and may be an effective remedy in those cases who have surgical contraindications. However, Sapylin is not side effect free. We suggest the dosage of Sapylin should be 0.1-0.2KE/kg/day and intervals should be over three days. It takes at least three days or longer for sapylin intraperitoneal injection to show the side effect. Saplin is also contraindicated for those patients who are allergic to penicillin, immunocompromised, with heart or kidney disease, rheumatism. A prospective multicenter randomized controlled study is also required to assess the safety and efficacy.

In recent years, the prognosis of CC has been improved since the development of medical technology and the nutrition interventions. Ergaz et al.^10^ evaluated the post-discharge clinical and developmental course of 11 neonates with CC, and concluded the recovery from chylothorax and future prognosis were dependent on the underlying etiology. Our patients were followed up from 6 months to two years and this progress will be continue.
